# The Effect of Packed Red Blood Cell Transfusion on Complete Blood Count and Hemoglobin Fractions in Adult Patients with Sickle Cell Disease: A Prospective Single-Center Cohort Study in Saudi Arabia

**DOI:** 10.3390/medsci14060668

**Published:** 2026-10-13

**Authors:** Rehab Yusuf Al-Ansari, Fatimah Salem Al Ayidh, Rayan Mansour Aljurbua, Mohammed Ibrahim AlJamily, Yassr Abdullah Alsubaie, Abdulelah Abdulrahman Almugahwi, Tawasoul Fadoul, Abdulrahman Hassan Halawi, Zechariah Jebakumar Arulanantham, Alexander Woodman

**Affiliations:** 1 Adult Hematology Unit, Internal Medicine Department, King Fahd Military Medical Complex, Dhahran 31932, Saudi Arabia; maljamily@yahoo.com; 2 College of Medicine, Imam Abdulrahman Bin Faisal University, Dammam 34212, Saudi Arabia; 3 Laboratory Department, King Fahd Military Medical Complex, Dhahran 31932, Saudi Arabia; fa6mah@hotmail.com (F.S.A.A.); ar.halawi@gmail.com (A.H.H.); 4 Internal medicine Department, King Fahd Military Medical Complex, Dhahran 31932, Saudi Arabia; aljurbua.rm@gmail.com (R.M.A.); iizyzoom@gmail.com (Y.A.A.); abdulelah.almugahwi@gmail.com (A.A.A.); tawassulmoh@hotmail.com (T.F.); 5 Vice Deanship of Postgraduate Studies and Scientific Research, Prince Sultan Military Collage of Health Sciences, Dhahran 31932, Saudi Arabia; zacbiostat@gmail.com; 6 Warwick Medical School, University of Warwick, Coventry CV4 7AL, UK; alexwoodman.ucla@gmail.com

**Keywords:** blood transfusion, complete blood count, high-performance liquid chromatography, hemoglobin fractions, sickle cell disease

## Abstract

**Background:** Sickle cell disease (SCD) is a genetic disease where a mutation in beta-globin results in an increased hemoglobin S percentage (Hgb S%), which is responsible for the crisis. The aim of this study was to determine the impact of each unit packed red blood cell (PRBC) simple transfusion on complete blood count (CBC) and high-performance liquid chromatography (HPLC) parameters in adult patients with SCD. **Methods:** This prospective cohort study was conducted between 29 September 2024, and 30 July 2025, among *n* = 65 SCD patients requiring hospitalization in the hematology department for blood transfusions. Medication, frequency of admission, and demographic information were obtained from the health informatics system. In addition, CBC, white blood cell (WBC), hemoglobin (Hgb), platelet (Plt), and HPLC data were obtained within 24 h before and after the PRBC transfusion. **Results:** Of *n* = 65 SCD patients, 87.7% matched the SS phenotype; 58.5% were females; the mean age was 27.23 years. Blood group O+ accounted for 50.8% of the patients, followed by A+ (33.8%). The mean Hgb values before and after transfusion were 7.16 (standard deviation (SD) 0.82) and 8.67 (SD 0.96), respectively (*p* < 0.001). The mean Hgb S% values before and after transfusion were 71.25 (SD12.19) and 55.97 (SD11.24), respectively (*p* < 0.001). The difference in the mean values of WBC, Hgb F% and Hgb A% (*p* < 0.001), as well as Hgb A2% (*p* = 0.006), changed significantly before and after blood transfusion. A negative correlation between body mass index (BMI) and changes in Plt count was found (*p* = 0.009). **Conclusions:** This study quantified the impact of each PRBC unit on CBC and HPLC parameters. While these findings may support strategies to reduce alloimmunization, this outcome was not evaluated and requires further study.

## 1. Introduction

Sickle cell disease (SCD) is a multisystem genetic disorder in which a mutation in beta globin leads to an increased percentage of hemoglobin S (Hgb S%), causing sickle cell crisis. This process results in a complex interaction of hemolysis (red blood cell destruction), chronic inflammation, and systemic vascular damage. Beyond its primary symptom of acute and chronic pain, SCD also profoundly affects multiple organs and systems throughout the body [1,2,3]. Neuropathic pain, acute pain crisis, chronic pain syndrome, hemolytic crisis, aplastic crisis, splenic sequestration crisis, hepatopathy, cardiopulmonary complications, especially pulmonary hypertension, acute chest syndrome and cardiomyopathy, as well as complications from opioids or other drugs and blood transfusions, have also been reported as symptoms of SCD [1,4,5]. Therefore, treating SCD requires attention to its complex pathophysiology and the subtle impact it has on comorbidities, which become increasingly common as people reach adulthood [1,2,3].

Additional evidence suggests that complications of SCD account for a significant proportion of deaths in the first two to three years of life [6]. Previously, treatment for SCD relied entirely on blood transfusions, both simple and exchange, to reduce a patient’s Hgb S% by diluting their blood with normal PRBC units [5,7]. Therapeutic blood transfusions have been used for a number of complications, including acute chest syndrome, stroke, sickle cell hepatopathy, splenic and hepatic sequestration, acute symptomatic anemia, and central retinal artery occlusion [7,8]. Nevertheless, information on the percentage reduction in Hgb S% levels in adults with SCD receiving PRBC units is limited. One retrospective study predicting Hgb S% levels after blood transfusion found that pretransfusion Hgb S% levels were insufficient for accurate quantification [9].

Sickle cell disease is particularly prevalent in sub-Saharan Africa, parts of India, the Caribbean, the Mediterranean region, and the Middle East. In Saudi Arabia, data from the national premarital screening program showed that 4.2% of screened adults were carriers of the sickle cell trait and 0.26% had SCD. The prevalence of SCD varies considerably across the Kingdom of Saudi Arabia, with the highest rates reported in the Eastern Province. The prevalence of sickle cell trait has been reported to range from 2% to 27%, and the prevalence of SCD may reach up to 2.6% in some regions [1,2].

Further evidence relating to this health condition suggests that mutations in the beta subunit of hemoglobin (HBB) can cause hemoglobin S (Hgb S), hemoglobin C (Hgb C), hemoglobin D (Hgb D), hemoglobin E (Hgb E), and hemoglobin O (Hgb O) to become aberrant forms of beta globin [3]. In the Eastern Province of Saudi Arabia, a mixed pattern of SCD and thalassemia (Hgb SB) is observed. These differences, which influence the severity and clinical presentation of the disease, are known as SCD phenotypes [3,4,5].

A significant knowledge gap remains regarding the quantitative effect of a single simple PRBC transfusion on Hb S% and other CBC and HPLC parameters in adults with SCD. Given the available data and remaining knowledge gaps, the primary objective of this study was to evaluate the effects of a single PRBC transfusion on CBC parameters and hemoglobin fractions measured by HPLC in patients with SCD in Eastern Saudi Arabia. Secondary objectives were to quantify the rate of decline in Hb S% following transfusion and to evaluate the association of patient characteristics, including ABO blood group and body mass index (BMI), with the magnitude of Hb S% reduction. We hypothesized that each PRBC unit would result in measurable changes in CBC and HPLC parameters and that the degree of Hb S% decline may vary according to patient-specific factors. Ultimately, the collected data may help to develop a strategic treatment plan aimed at limiting volume and iron overload in the preoperative, postoperative, or perinatal stages in patients requiring blood transfusion to lower Hgb S%.

## 2. Materials and Methods

### 2.1. Study Design and Setting

This prospective observational cohort study was conducted at a single tertiary hospital in the Eastern Province of Saudi Arabia between 29 September 2024 and 30 July 2025. The single tertiary hospital where the study was initiated serves military personnel, their families, health workers and other beneficiaries in the Eastern Province, providing a wide range of opportunities for data collection. The study included 65 patients with SCD who were admitted to the regular beds in the hematology department and required simple blood transfusion rather than exchange transfusion. In our institute, the diagnosis of SCD is established based on clinical history, followed by a sickling test as an initial screening tool. Positive cases are subsequently confirmed by hemoglobin electrophoresis and HPLC.

The primary outcome was to evaluate the effects of a single PRBC transfusion on CBC parameters and hemoglobin fractions measured by HPLC in patients with SCD. These included hemoglobin concentration, Hb S%, HbA%, and other relevant hematologic parameters. Secondary outcomes included the rate of decline in Hb S% after transfusion and the association between the magnitude of Hb S% reduction and patient-related factors, including ABO blood group and body mass index (BMI).

All transfused PRBC units were leukoreduced according to institutional blood bank policy, with a typical volume of approximately 450 mL per unit. Data regarding the number of transfused units were recorded for each participant. Information on irradiation, extended antigen matching, and other transfusion modifications was not routinely available and was therefore not included in the analysis. The transfused units were prepared according to standard blood bank procedures at our institution.

### 2.2. Study Sample

The sample size was estimated using a descriptive-study approach based on the anticipated number of adult patients with SCD requiring PRBC transfusion at our institution during the study period. Hematologists at the tertiary care center in the Eastern Province of Saudi Arabia, where the study was conducted, provide care for approximately 380 adult patients with SCD annually, of whom 153 were hospitalized in 2023 and 48% received transfusion therapy. Based on these figures, the required sample size was estimated at 64 ± 5 participants (range 59–69). Because of the limited prospective data available on the quantitative effect of individual PRBC units on changes in specific hemoglobin fraction parameters measured by CBC and HPLC in adults with SCD, a formal power calculation based on paired continuous outcomes was not feasible. Therefore, the sample size should be considered exploratory and feasibility-based.

### 2.3. Eligibility Criteria

Inclusion criteria: Adult patients with SCD of any phenotype who met the eligibility criteria for therapy established by the research institute were eligible to participate in the study. Patients must be at least 14 years of age (in accordance with the age restrictions of the institution: the age of majority begins at 14 years). Patients requiring hospitalization in a regular ward and simple blood transfusion for medical reasons (e.g., hemolysis associated with active sickle cell crisis) were also included.

Exclusion criteria: Patients with sickle cell trait (SCT), patients without blood transfusion, pregnant women, patients with active bleeding from any cause, and critically ill patients were excluded from the study. In addition, patients who did not meet the criteria for admission to the hematology department, as well as pediatric patients (in accordance with the age restrictions of the institution, namely those under 14 years old), were not included in this study.

Eligible patients were recruited consecutively based on the study inclusion and exclusion criteria. The recruitment process is summarized in the flow diagram presented in Supplementary Material Figure S1.

### 2.4. Procedures

Ethical approval was obtained from the Eastern Province Armed Forces Hospital Institutional Review Board (AFTER-IRB-2024-038, 24 September 2024). Patients were provided with written informed consent to participate in the study, which included consent for laboratory tests before and after PRBC transfusion, including a new CBC test and HPLC 24 h before and after the blood transfusion. Although adulthood in our institution is defined as ≥14 years of age, participants aged 14 to 17 years were required to provide assent, with informed consent obtained from their parent or legal guardian prior to participation in the study. A complete data set, including demographic data, patient medical records, and test results, was provided by the health informatics system. Data from the medical informatics system were compiled into an Excel spreadsheet and submitted to the principal investigator (PI). This study adhered to the principles of the Declaration of Helsinki. Generative artificial intelligence (GenAI) has not been used in this paper.

### 2.5. Data Collection

Data on age, gender, underlying comorbidity, SCD phenotyping, frequency of admission (<3 times/year versus ≥3 times/year), BMI, use of Hydroxyurea, and history of alloimmunization were collected. Admission frequency was defined as the number of hospital admissions related to SCD experienced by a patient during the study period. In addition, simple blood transfusion laboratory parameters such as CBC (including WBC, RBC, and Plt) and HPLC (including Hgb S%, Hgb A%, Hgb A2%, and Hgb F%) were collected at baseline and post-transfusion. Blood grouping and the amount of blood transfused (ML) were also recorded. A patient was included in the study only once, even if in certain situations a blood transfusion was required upon rehospitalization.

### 2.6. Instrumentation

A CBC was performed as part of daily laboratory testing for monitoring patients with SCD in ongoing crises. If a blood transfusion was required, a baseline blood test using HPLC was included. Blood grouping was performed only if no baseline blood grouping or cross-matching tests had been performed prior to the transfusion. At 24 h post-transfusion, another set of CBC and HPLC tests was taken for re-evaluation and research purposes.

The Alinity Hq instrument was used to examine the CBC. In addition, ABO blood group was automatically determined at the local center and other centers in Saudi Arabia using a microplate method that essentially determined the presence of homologous antigens (anti-A and anti-B) [10]. The eluted samples were analyzed using an HPLC from Bio-Rad Laboratories in Hercules, CA. HPLC was used to determine percent and percentage hemoglobin variations (Hgb SS, Hgb SB, Hgb SC, Hgb SD, Hgb SE, Hgb SO, and Hgb S trait). Each hemoglobin variant has a distinct retention duration. The interval between injecting the sample and the peak of a hemoglobin peak is referred to as retention time [11].

### 2.7. Statistical Analysis

An Excel worksheet was used for the database. The Social Science Statistical Package (SPSS version 28, IBM Corp., Armonk, NY, USA) was used to analyze the patients’ data for baseline features, associations, and correlations. *T*-test analysis was used to determine the relationship between PRBC transfusion, CBC, HPLC, blood grouping, and BMI. Descriptive statistics were calculated using categorical variables’ frequency (*n*) and percentage (%). The Mean, Standard Deviation (SD), and Std. Error were applied to analyze the quantitative (numerical) variables (Tables 1–3 and Tables S1–S3). A paired *t*-test was used for the analysis presented in Table 2. An independent-samples *t*-test was used for the analyses presented in Supplementary Tables S1–S3. Pearson correlation analysis was used in Table 3.

Due to the relatively small sample size and the non-normal distribution of the outcome variable, the Mann–Whitney U test was considered more appropriate than the independent-samples *t*-test. This non-parametric approach was used to assess whether there was a statistically significant difference between the two independent groups. A *p*-value of less than or equal to 0.05 with a 95% confidence interval was deemed statistically significant. No missing data were observed for the variables analyzed, and therefore no imputation procedures were required.

## 3. Results

A total of 214 patients were assessed for eligibility, of whom 65 were included in the final analysis (Supplementary Figure S1). No missing data were identified for the principal study variables; and, therefore, all enrolled participants were included in each analysis. Descriptive statistics showed that the mean age of the participants was 27.23 years, with the majority being female (58.5%) (Table 1a). Approximately 63.1% of patients were taking Hydroxyurea, while 36.9% were not (due to side effects or a history of allergies and other complications). Additionally, ABO blood grouping was performed; most patients had group O+ (50.8%), followed by group A+ (33.8%) (Table 1b).

The Hgb SS phenotype was detected in 87.7% of participants, and the Hgb SB phenotype was detected in 12.3%. Although most patients had SCD alone, 6.6% also had glucose-6-phosphate dehydrogenase (G6PD) deficiency and 9.8% had concomitant asthma. Alloimmunization was detected in 24.6% of patients. Lastly, the mean BMI was 22.17 (SD 4.84) with a range of 14.68 to 35.92 (Table 1b).

Table 2 presents the CBC and HPLC values before and after the transfusion of one unit of PRBC containing approximately 450 milliliters of blood. The mean Hgb value before the transfusion was 7.16 (SD 0.82), and after the transfusion it was 8.67 (SD 0.96). Each unit of PRBC transfusion resulted in a substantial increase in Hgb of 1.51 g/dL (*p* < 0.001). The mean WBC before transfusion was 12.67 (SD 5.27), and after transfusion decreased to 11.12 (SD 4.09). Each unit of PRBC transfusion significantly reduced the WBC differential by 1.55 × 10^9^/L (*p* < 0.001). Nevertheless, there was no significant effect on the mean Plt count (*p* = 0.35) (Table 2).

Further results presented in Table 2 showed that the mean Hgb S% level was 71.25 (SD 12.19) before transfusion and 55.97 (SD 11.24) after transfusion, with a significant decrease of 15.28% (*p* < 0.001). Moreover, the mean difference in Hgb A% level before and after blood transfusion significantly increased by 16.90% (*p* < 0.001). In addition, there was a significant difference in Hgb A2% level before and after blood transfusion (*p* = 0.006). Moreover, the mean Hgb F% showed a reduction of 1.33%, which was statistically significant (*p* < 0.001) (Table 2).

Further analysis of this study examined the relationship between the difference in mean values of CBC parameters before and after blood transfusion depending on the frequency of hospitalizations and blood grouping. As shown in Supplementary Material Table S1a,b, the results before and after PRBC transfusion for both CBC and HPLC parameters were not significant (*p* > 0.05). Due to the small sample size, blood groups were divided into O and non-O groups. The Mann–Whitney test showed no significant difference between blood groups and the change in CBC and HPLC results before and after blood transfusion (*p* > 0.05) (Table S1b). Furthermore, differences in CBC and HPLC parameters before and after PRBC transfusion did not compare with Hydroxyurea use or annual transfusion rate (*p* > 0.05) (Tables S2 and S3).

**Figure 1:**
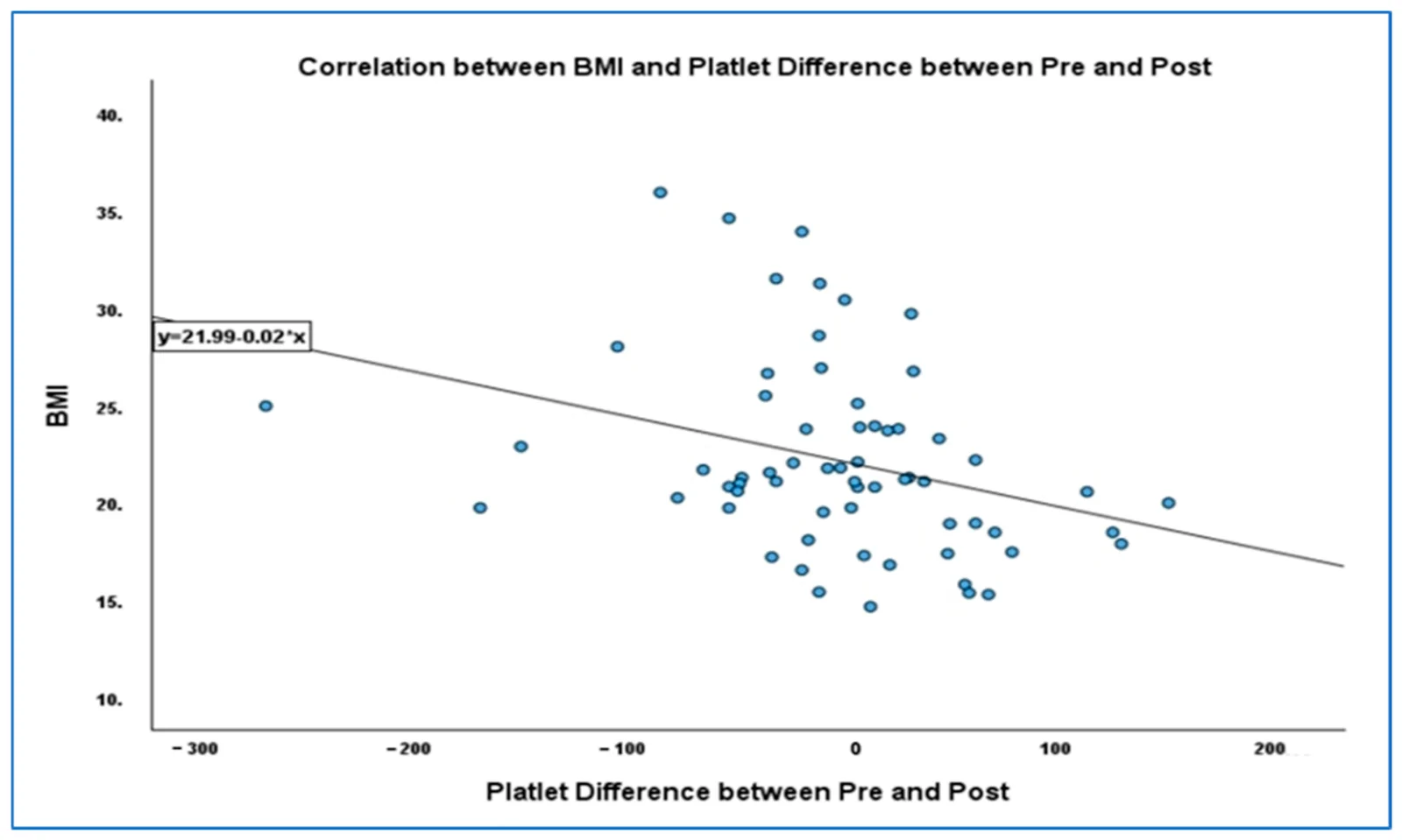
Association between body mass index (BMI) and changes in platelet count. * Abbreviation: BMI = body mass index, Plt = platelet.

Table 3 presents the Pearson correlation analysis between BMI and selected hematological parameters, including Hgb, Plt, WBC, Hgb S%, Hgb A%, Hgb A2%, and Hgb F%. Among the variables examined, only Plt count demonstrated a statistically significant correlation with BMI (*p* < 0.05) (Table 3). The correlation coefficient (*r* = −0.32) indicated a weak-to-moderate negative correlation, suggesting that as BMI increased, Plt count tended to decrease. The coefficient of −0.32 reflected the strength and direction of the linear relationship. Figure 1, representing a Scatter plot (diagram), showed a linear relationship between BMI and changes in Plt counts. No statistically significant correlations were observed between BMI and the remaining hematological parameters (*p* > 0.05).

## 4. Discussion

This study was initiated to better understand and acquire specific knowledge regarding the impact of PRBC transfusion on CBC and HPLC in Saudi patients with SCD.

The findings demonstrated a significant reduction in Hgb S%, WBC count, and Hgb A2% following each unit of PRBC transfusion. In addition, a significant negative association was observed between BMI and the change in platelet count (*p* = 0.009). These results suggest that PRBC transfusion effectively modifies hematological and hemoglobin fraction parameters in patients with SCD, with transfusion response potentially influenced by patient-specific factors such as BMI.

Sickle cell disease is characterized by the formation of sickle-shaped red blood cells that obstruct blood flow and impair oxygen delivery, leading to progressive organ damage and other disease-related complications. These consequences are caused by a genetic mutation that leads to the formation of abnormal hemoglobin, known as Hgb S%, whose levels must be reduced to treat SCD. This can be achieved through blood transfusions (simple or exchange) or drugs such as Hydroxyurea [12].

Descriptive analysis data showed that females predominated among the 65 patients included in the current cohort. However, currently available information does not reflect differences in gender or ethnicity among Saudi SCD patients [13,14]. This discrepancy may be due to the specificity of the current study sample, which included patients requiring blood transfusion, as well as the fact that females of reproductive age are more likely to experience a decrease in hemoglobin levels.

Furthermore, 50.8% of patients had blood type O+, which is consistent with previous studies in the Saudi population [15,16]. Overall, the distribution of ABO blood types in Saudi residents with SCD is similar to that in the national population, with type O being the most common [13]. However, in this study, when analyzing the differences in CBC and HPLC parameters before and after PRBC transfusion in patients with and without blood group O, no association was found (*p* > 0.05). This finding is consistent with a previous study by Uter et al. (2021), which showed that the rate of decline in Hgb S% in SCD does not correlate with ABO blood type [17]. Instead, the researchers emphasized that donor unit volume, antigen matching approach, relative Hgb S% pre-transfusion, and transfusion method (simple or exchange) all influence the reduction in Hgb S% [17].

Further findings from this study showed an alloimmunization rate of 24.6%, with further phenotyping revealing no significant differences. This number is considered high due to the frequent need for blood transfusions in patients with prior hemolysis or complications such as sickling complications. In a previous local study, the risk of alloimmunization was primarily due to non-ABO antigens, such as the e antigen, which accounted for over 90% of alloimmunization causes [15]. In order to reduce the risk of alloimmunization in cases of hemoglobinopathies such as SCD, Halawani et al. (2022) studied the frequency of alloimmunization in the southern region of Saudi Arabia, Jizan [16]. The findings, echoed in other studies, highlight the need for expanded phenotyping to include ABO, RH (C, D, E, c, e), KEL (K/k and Kp (a, b)), Fy (a, b), and Jk (a, b) phenotypes [15,16,18]. Accordingly, extended phenotyping of RBCs and genotype matching are critical to limit alloimmunization in SCD, particularly in Saudi Arabia, where the risk of blood transfusion is high and national guidelines are lacking. The position paper by AlHumaidan et al. (2025) entitled “Position Paper on the Management of SCD in Saudi Arabia: Understanding Disease Landscape, Identifying Challenges, and Exploring Opportunities in Transfusion Therapies” acknowledges significant practice variability and strongly supports evidence-based national transfusion guidelines and standardized extended antigen matching to improve patient outcomes [13]. This procedure was implemented in accordance with the 2020 American Society of Hematology (ASH) guidelines for blood transfusion in patients with SCD [13,19].

SCD in the Kingdom of Saudi Arabia is characterized by a range of hemoglobin variations, including Hgb D-Punjab, Hgb C, Hgb E, Hgb F-Dammam, Hgb O-Arab, Hgb S, Hgb-Riyadh, Hb-Handsworth, and Hgb-Setif, indicating regional genetic heterogeneity and historical migratory patterns [20]. In local practice, HPLC is used to quantify hemoglobin fractions such as A2, A, C, E, F, D, and S percentages. This technique is considered the gold standard for diagnosing SCD, distinguishing between SCD and SCT, confirming phenotypes, monitoring Hgb S%, and assessing blood transfusion effectiveness [21]. Thus, the current cohort highlights the extent to which the HPLC parameter affected by each RBC unit is reported, with a close time interval when monitoring the outcome (24 h before and after), which has not been sufficiently studied previously [8]. A study by Uter et al. (2021) roughly explains how donor RBC exchange reduces Hgb S% and increases HbA%, as well as how major changes in HPLC occur after exchange transfusion in the context of the COVID-19 pandemic [17]. However, this is not consistent with the methodology used in this study, which is primarily based on predicting changes in laboratory parameters after a simple blood transfusion rather than after an exchange transfusion.

This study also showed a significant decrease in Hgb S% between the pre-PRBC transfusion period (minimum pre-transfusion value was 40.9, maximum 88.7, and mean 71.256) and post-transfusion period (minimum post-transfusion value was 29.6, maximum 79.0, and mean 55.974) (*p* < 0.001). The reduction in the mean Hgb S% before and after transfusion was almost 15% ± 5 per unit. This is somewhat contrary to the study by Mathur et al. (2018), who highlighted that Hgb S% levels decreased by approximately 7% after a simple blood transfusion in 24 children with SCD [9]. The findings may aid in targeted management of blood transfusions and development of transfusion formulas to minimize the amount of transfusion and ultimately prevent iron overload and/or alloimmunization. However, published data on the specific hematological and hemoglobin fraction changes associated with individual PRBC transfusions are scarce. The available pediatric studies are cited to illustrate this knowledge gap rather than for direct comparison with our adult cohort.

This study also found a significant increase in Hgb levels and Hgb A% (*p* < 0.001). Overall, in a patient without SCD, one unit of PRBC would be expected to increase Hgb by 1 g/dL [22]. However, the rate of increase in Hgb levels may depend on factors such as the type of blood transfusion, patient weight, volume, and amount of irradiated blood, as well as the recovery and survival of transfused red blood cells [23]. In a previous study by Mathur et al. (2018), the mean Hgb level before transfusion increased from 8.2 g/dL to 10.2 g/dL after transfusion, with a difference of approximately 2 g/dL depending on the age of the children [9]. This is in contrast to the results of the current study, in which the mean Hgb level increased by almost 1.5 g/dL and the Hgb A% increased by approximately 17%. Additional data suggest that a PRBC transfusion presumably dilutes Hgb S by adding red blood cells containing normal Hgb A. Based on the current study results, an inverse 1:1 relationship is observed between the decrease in Hgb S and the increase in Hgb A after each 450 mL transfusion.

No association was found between pre- and post-treatment differences in CBC and HPLC values and the use of Hydroxyurea, history of blood transfusions, or hospitalization rate (as indicators of disease severity). Although Plt counts did not differ significantly before and after transfusion (*p* = 0.35), an inverse relationship was observed between BMI and Plt counts after transfusion (higher BMI = smaller changes in Plt count). Because PRBC transfusions do not contain Plts, they can produce dilutional thrombocytopenia [24]. SCD patients frequently exhibit baseline thrombocytosis due to functional asplenia. A slight decrease in Plt count after transfusion is expected but is not clinically significant [25]. Fluctuations in Plt count are related to Plt transfusion, not RBC transfusion. This supports the idea that PRBC transfusion does not increase Plt count [26].

Finally, regarding the influence of BMI on CBC parameters, earlier studies reported a positive correlation with WBC and Plt counts, but a negative correlation with Hgb in young patients without SCD [27,28]. In SCD, where baseline leukocytosis and thrombocytosis are already present due to hemolysis and functional asplenia, excess adipose tissue is expected to enhance the increase in leukocyte and Plt counts, although Hgb levels are primarily determined by disease severity rather than BMI. Thus, obesity in SCD is a novel modulator of CBC parameters, particularly leukocyte and Plt counts, through additive inflammatory and metabolic pathways [29]. However, no study using tunnel access explains precisely how BMI affects CBC values in patients with SCD.

Overall, the results of this study may contribute to the existing literature by exploring a topic that has received little attention so far and should serve as a basis for further research, improvement projects and/or development of guidelines aimed at improving blood transfusion practices in patients with SCD.

Several strengths should be acknowledged. Its prospective design and within-patient pre/post transfusion assessment allowed direct evaluation of transfusion-related changes while minimizing inter-individual variability. In addition, laboratory measurements were obtained over a short, standardized interval, reducing the potential influence of time-dependent confounding factors. Finally, the study assessed the effects of simple PRBC transfusion in an adult SCD population, a group that remains relatively underrepresented in the literature, particularly in Saudi Arabia.

This study has several limitations that require further consideration. First, it was conducted at a military center with a limited number of participants. Second, the small sample size is concerning due to the lack of funding for a prospective cohort study. Third, as with any observational study, heterogeneity in patient characteristics, clinical history, vaccination and treatment exposure may have influenced the observed outcomes. Fourth, the single-center design and modest sample size may limit the generalizability of the results. Fifth, the study population consisted of hospitalized patients requiring transfusion, which may not be representative of all patients with SCD. Also, residual confounding cannot be excluded despite the prospective design. In addition, multiple statistical comparisons were performed, increasing the possibility of type I error. Furthermore, measurement variability and differences in transfusion-related factors may have influenced the observed changes. Finally, the study focused on short-term laboratory outcomes and did not assess clinical outcomes or longer-term effects of transfusion.

The generalizability of these findings should be considered in light of the study population and setting. The results were derived from a single tertiary care center in Eastern Saudi Arabia and primarily reflect young adult patients with Hgb SS who required inpatient simple transfusion. Consequently, the findings may not be directly applicable to other SCD populations, outpatient settings, patients receiving exchange transfusion, pediatric populations, pregnant women, or healthcare systems with different patient characteristics and transfusion practices.

Future research should focus on validating these findings in larger, multicenter cohorts. Studies evaluating predictors of post-transfusion Hgb S%, including patient characteristics, donor and transfusion unit factors, and SCD genotype, may help optimize transfusion strategies. In addition, assessment of clinically relevant outcomes and transfusion-related complications is needed to better define the long-term impact of PRBC transfusion in patients with SCD. Future research should also investigate post-transfusion hemolytic complications, including hyperhaemolysis and bystander hemolysis. Incorporating hemolysis markers, alloantibody and direct antiglobulin test (DAT) evaluation, as well as detailed transfusion histories, may improve understanding of transfusion-related risks, particularly in previously alloimmunized patients [30].

## 5. Conclusions

This study demonstrated the impact of a single PRBC transfusion on CBC and HPLC parameters in patients with SCD. Significant changes were observed in several hematological indices, including a reduction in Hgb S%, following transfusion. These findings contribute to understanding the short-term laboratory effects of PRBC transfusion and may help guide future studies aimed at optimizing transfusion practices. The observed changes in WBC counts may also warrant further investigation into distinguishing sickle cell crisis-related leukocytosis from infection. Further multicenter studies are needed to validate these findings and evaluate their clinical implications and long-term outcomes. Additionally, future research should investigate whether alloimmunization status influences hematological and hemoglobin fraction changes following PRBC transfusion.

## Figures and Tables

**Table 1:** (**a**). Age and BMI mean and Standard Deviation. (**b**). Demographic and Clinical Characters.

**(a)**					
**Covariates**	**N**	**Minimum**	**Maximum**	**Mean**	**Std. Deviation**
Age	65	14	55	27.23	9.172
Weight	65	21.5	98	53.72	14.26
Height	65	102	180	155.57	13.14
BMI *	65	14.68	35.92	22.17	4.84
**(b)**					
**Demographic Characteristics**		**Frequency (N)**		**Percentage (%)**	
**Gender**					
Male		27		41.5	
Female		38		58.5	
**HU Use**					
Yes		41		63.1	
No		24		36.9	
**ABO group**					
A+		22		33.8	
AB+		2		3.1	
B-		1		1.5	
B+		4		6.2	
O-		3		4.6	
O+		33		50.8	
**SCD phenotype**					
SB		8		12.3	
SS		57		87.7	
**Comorbidity**					
Bronchial Asthma		6		9.8	
CKD		2		3.3	
Delayed Puberty		1		1.6	
Epilepsy		2		3.3	
G6PD		4		6.6	
No		50		76.9	
**Allo-Immunization**					
Yes		16		24.6	
No		49		75.4	

* BMI = body mass index. Key words: CKD = chronic kidney disease, G6PD = Glucose-6-phosphate dehydrogenase deficiency, HU = Hydroxyurea, SCD = sickle cell disease.

**Table 2:** Evaluation of CBC, HPLC laboratory parameters pre- and post-transfusion.

Covariates	N	Minimum	Maximum	Mean	Calculated Difference in Mean (SD)	Std. Deviation (SD)	Pre/Post-Transfusion Confidence Interval 95% (CI)	Mean Pairs Confidence Interval 95% (CI)	Std. Error Mean	*T*-Test	*p*-Value
pre-Hgb	65	5.37	9.70	7.16	Increment by 1.51 g/dL (0.14)	0.82	6.96–7.36	1.476–1.544	0.10196	17.12	<0.001 *
post-Hgb	65	6.79	11.40	8.67		0.96	8.90–8.44		0.11943		
pre-Plt	65	63.2	878.0	408.66	Reduction By 7.83 × 10^9^/L (0.1)	180.23	364.83–452.49	7.806–7.854	22.3552	0.94	0.35
post-Plt	65	62.8	807.0	400.83		180.13	357.03–444.63		22.3434		
pre-WBC	65	4.83	28.60	12.67	Reduction By 1.55 × 10^9^/L (1.18)	5.27	11.39–13.95	1.263–1.837	0.66324	4.23	<0.001 *
post-WBC	65	4.46	25.70	11.12		4.09	10.13–12.11		0.51169		
pre-Hgb S%	65	40.9	88.7	71.25	Reduction By 15.28% (0.95)	12.19	68.29–74.21	15.049–15.511	1.5492	17.01	<0.001 *
post-Hgb S%	65	29.6	79.0	55.97		11.24	53.24–58.70		1.4282		
pre-Hgb A%	65	4.0	51.2	18.45	Increment by 16.90% (1.31)	12.77	15.35–21.55	16.581–17.219	1.6090	18.03	<0.001 *
post-Hgb A%	65	13.7	60.2	35.36		11.458	32.57–38.15		1.4436		
pre-Hgb A2%	65	1.0	6.7	3.35	Reduction By 0.21% (0.23)	1.089	3.09–3.62	0.154–0.266	0.1372	2.84	0.006 *
post-Hgb A2%	65	1.6	6.7	3.13		0.8852	2.91–3.35		0.1115		
pre-Hgb F%	65	0.0	25.2	6.63	Reduction By 1.33% (8.35)	5.40	5.30–7.98	−0.832–3.232	0.6703	0.63	<0.001
Post-Hgb F%	65	0.00	20.9	5.30		4.13	4.28–6.33		0.5128		
calculated Hgb S% reduction	65	0.2	32.0	15.28	-	6.29	13.75–16.81		-	-	-

Key words: Hgb = hemoglobin, Plt = platelet, WBC = white blood cell. * Significant at 95%.

**Table 3:** Correlation between Mean changes in CBC parameters and BMI.

Dependent Variable	Independent Factors (Covariates)						
BMI	Hgb Difference Between Pre and Post	Plt Difference Between Pre and Post	WBC Difference Between Pre and Post	Hgb S% Difference Between Pre and Post	Hgb A% Difference Between Pre and Post	Hgb A2% Difference Between Pre and Post	Hgb F% Difference Between Pre and Post
Pearson Correlation	−0.088	−0.323 **	−0.137	−0.143	0.191	0.033	−0.196
*p*-value	0.488	0.009 *	0.283	0.270	0.137	0.797	0.126
N	65	65	65	65	65	65	65

Key words: Hgb = hemoglobin, Plt = platelet, WBC = white blood cell. * Significant at 95%; ** significant correlation.

## Data Availability

The original contributions presented in this study are included in the article/Supplementary Material. Further inquiries can be directed to the corresponding author.

## Supplementary Materials

The following supporting information can be downloaded at: https://www.mdpi.com/article/10.3390/medsci14060668/s1, Figure S1: Flow Diagram Highlight the Steps of Patients Recruitment; Table S1: (a) Comparisons between changes in Mean of CBC parameters with a unite of PRBC transfusion in relation to the frequency of admission and blood grouping; (b) Comparisons between changes in Mean of CBC parameters with a unite of PRBC transfusion in relation to the blood grouping; Table S2: Comparisons between Mean of CBC parameters difference between pre and post transfusion in relation to Hydroxyurea use; Table S3: Comparisons between Mean of CBC parameters difference between pre and post transfusion vs. frequency of blood transfusion per year.
