# Supplementary material for: The Effect of Packed Red Blood Cell Transfusion on Complete Blood Count and Hemoglobin Fractions in Adult Patients with Sickle Cell Disease: A Prospective Single-Center Cohort Study in Saudi Arabia
Source: Med Sci (Basel). 2026 Oct 13;14(6):668. doi: 10.3390/medsci14060668 (PMC13707288; doi:10.3390/medsci14060668)
Supplement: Supplementary file 1 [file medsci-14-00668-s001.zip › medsci-4562682-supplementary.pdf]

medical sciences

MDPI

The Effect of Packed Red Blood Cell Transfusion on Complete Blood Count and Hemoglobin Fractions in Adult Patients with Sickle Cell Disease: A Prospective Single-Center Cohort Study in Saudi Arabia

Figure S1. Flow Diagram Highlight the Steps of Patients Recruitment.

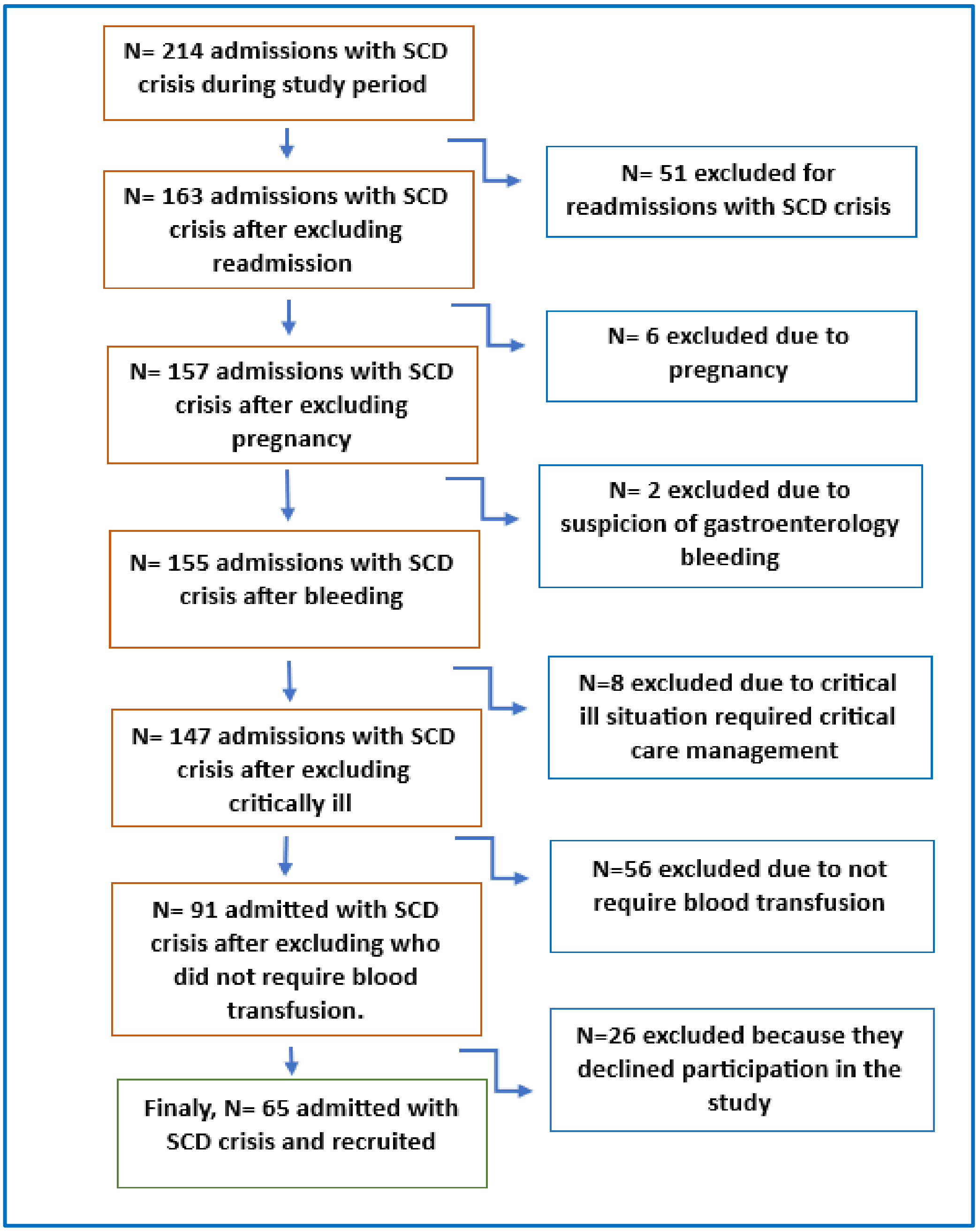

Abbreviations: N; patients number, SCD; Sickle Cell Disease.

Table S1: (a) Comparisons between changes in Mean of CBC parameters with a unite of PRBC transfusion in relation to the frequency of admission and blood grouping.

	Admission per last year	N	Mean of CBC parameter changes	Std. Deviation (SD)	Confidence Interval 95% (CI)	Std. Error Mean	T – test	P - value
**Mean of Hgb difference between pre and post**	Less than 3	23	1.69	.67	1.40 -1.98	.14117	1.55	0.13
	3 or More than	42	1.41	.71?	1.19 – 1.63	.11067		
**Mean of Plt difference between pre and post**	Less than 3	23	3.32	63.67	24.21-30.85	13.27683	1.00	0.33
	3 or More than	42	-13.93	69.23	7.63-35.49	10.68343		
**Mean of WBC difference between pre and post**	Less than 3	23	-1.99	2.56	0.88-3.10	.54714	0.78	0.45
	3 or More than	42	-1.39	3.24	0.38-2.40	.50001		
**Mean of Hgb S % difference between pre and post**	Less than 3	23	-14.89	6.94	11.89-17.89	1.44907	0.34	0.73
	3 or More than	42	-15.51	6.81	13.39-17.63	1.09119		
**Mean of Hgb A % difference between pre and post**	Less than 3	23	16.67	7.38	13.48-19.86	1.54083	0.19	0.85
	3 or More than	42	17.04	7.56	14.69-19.39	1.19611		
**Mean of Hgb A2 % difference between pre and post**	Less than 3	23	-.35	.64	0.07-0.63	.13496	1.36	0.18
	3 or More than	42	-.14	.58	0.04-0.32	.09232		
**Mean of Hgb F % difference between pre and post**	Less than 3	23	-1.44	1.19	0.93-1.95	0.25	-0.73	0.47
	3 or More than	42	-1.13	1.88	0.54-1.72	0.29		

Abbreviation: Hgb= hemoglobin, Plt = platelet, WBC = white blood cell.

Table S1: (b) Comparisons between changes in Mean of CBC parameters with a unite of PRBC transfusion in relation to the blood grouping.

	Blood Grouping	N	Median of Laboratory value changes pre and post transfusion	Inter Quartile Range	Mann-Whitney U test / T-test	P - value
**Median of Hgb**	Non-O Blood Group	29	1.33	1.08	401.00	0.11
	O Blood Group	36	1.65	0.81		
**Median of Plt**	Non-O Blood Group	29	-14.80	56.00	480.00	0.58
	O Blood Group	36	1.000	78.00		
**Median of WBC**	Non-O Blood Group	29	-2.42	3.94	430.500	0.32
	O Blood Group	36	-1.20	2.80		
**Median of Hgb S %**	Non-O Blood Group	29	-13.05	10.53	360.500	0.11
	O Blood Group	36	-16.10	8.30		
**Median of Hgb A %**	Non-O Blood Group	29	14.30	11.47	380.000	0.13
	O Blood Group	36	17.70	9.40		
**Median of Hgb A2 %**	Non-O Blood Group	29	-0.15	0.65	488.000	0.98
	O Blood Group	36	-0.20	0.50		
**Median of Hgb F %**	Non-O Blood Group	29	-0.60	0.90	392.50	0.06
	O Blood Group	36	-1.10	3.10		

Abbreviation: Hgb= hemoglobin, Plt = platelet, WBC = white blood cell.

Table S2. Comparisons between Mean of CBC parameters difference between pre and post transfusion in relation to Hydroxyurea use.

	Hydroxyurea use	N	Mean of CBC parameter changes	Std. Deviation (SD)	Confidence Interval 95% (CI)	Std. Error Mean	T – test	P - value
**Mean of Hgb difference between pre and post**	Yes	41	1.47/	.73	1.24- 1.70	.11489	0.46	0.65
	No	24	1.56	.67	1.28 -1.84	.13862		
**Mean of Plt difference between pre and post**	Yes	41	-14.40	76.07	9.60 – 38.40	11.88127	1.03	0.31
	No	24	3.40	48.38	17.04-23.84	9.87739		
**Mean of WBC difference between pre and post**	Yes	41	-1.29	3.15	0.30 -2.28	.49964	1.05	0.30
	No	24	-2.11	2.75	0.95 – 3.27	.56232		
**Mean of Hgb S% difference between pre and post**	Yes	41	-15.10	7.41	12.76 –17.44	1.17254	0.28	0.78
	No	24	-15.60	5.70	13.20 –18.00	1.21705		
**Mean of Hgb A% difference between pre and post**	Yes	41	16.80	7.94	14.30 -19.30	1.25546	0.14	0.89
	No	24	17.07	6.65	14.26-19.88	1.38829		
**Mean of Hgb A2% difference between pre and post**	Yes	41	-.19	.58	0.01-0.37	.09201	0.45	0.65
	No	24	-.26	.67	0.02-0.54	.13996		
**Mean of Hgb F% difference between pre and post**	Yes	41	-1.49	1.95	0.88 -2.10	0.30	-1.69	0.096
	No	24	-0.83	1.09	0.37-1.29	0.22		

Key words: WBC = white blood cell, Hgb= hemoglobin, Plt = platelet.

Table S3. Comparisons between Mean of CBC parameters difference between pre and post transfusion vs frequency of blood transfusion per year:.

	Blood transfusion per last year	N	Mean of CBC parameter Changes	Std. Deviation (SD)	Confidence Interval 95% (CI)	Std. Error Mean	T – test	P - value
**Mean of Hgb difference between pre and post**	Less than 3	29	1.56	.69	1.30 – 1.82	.12814	0.54	0.59
	3 or More than	36	1.46	.73	1.21 -1.71	.12240		
**Mean of Plt difference between pre and post**	Less than 3	29	-12.71	77.85	16.93 – 42.35	14.45720	0.52	0.60
	3 or More than	36	-3.90	58.35	15.84 – 23.64	9.72644		
**Mean of WBC difference between pre and post**	Less than 3	29	-1.89	3.13	0.70 – 3.08	.59265	0.69	0.50
	3 or More than	36	-1.36	2.94	0.37 – 2.35	.49136		
**Mean of Hgb S% difference between pre and post**	Less than 3	29	-15.04	6.69	12.49 - 17.59	1.24323	0.25	0.80
	3 or More than	36	-15.48	7.01	13.11 - 17.85	1.22113		
**Mean of Hgb A% difference between pre and post**	Less than 3	29	16.87	7.16	14.14 - 19.60	1.33054	0.03	0.97
	3 or More than	36	16.93	7.78	14.30 - 19.56	1.33431		
**Mean of Hgb A2% difference between pre and post**	Less than 3	29	-.14	.58	0.08 -0.36	.10880	0.85	0.40
	3 or More than	36	-.27	.63	0.06 -0.48	.10894		
**Mean of Hgb F% difference between pre and post**	Less than 3	29	-1.33	1.54	0.74 -1.92	0.29	-0.37	0.71
	3 or More than	36	-1.18	1.79	0.57 -1.79	0.30		

Abbreviation: Hgb= hemoglobin, Plt = platelet, WBC = white blood cell.
